# Allylthioketone Mediated Free Radical Polymerization of Methacrylates

**DOI:** 10.3390/polym9110608

**Published:** 2017-11-13

**Authors:** Feng Zhong, Liang Qiu, Chun-Yan Hong, Cai-Yuan Pan

**Affiliations:** 1 CAS Key Laboratory of Soft Matter Chemistry, Department of Polymer Science and Engineering, University of Science and Technology of China, Hefei 230026, China; adzhf@mail.ustc.edu.cn; 2 Institute of Biophysics, Hebei University of Technology, Tianjin 300401, China; 2016035@hebut.edu.cn

**Keywords:** allylthioketone, controlled radical polymerization, livingness, electron paramagnetic resonance, MALDI-TOF MS

## Abstract

By combination of high trapping free radical efficiency of the thioketone and resonance of the allylic radical, a new type of mediating agent, 1,3,3-triphenylprop-2-ene-1-thione (TPPT) has been successfully synthesized, and then is used to study controlled free radical polymerization of methacrylates. Very stable TPPT radicals at the end of poly(methyl methacrylate) (PMMA) are detected in the polymerization of MMA using TPPT and AIBN as the control agent and initiator. The MALDI-TOF MS spectra are used to identify terminal groups of the resultant poly(glycidyl methacrylate) (PGMA), and major component of the obtained polymer has the structure, (CH_3_)_2_(CN)C-PGMA-C_7_H_9_O_3_. Chain extension reaction tests ascertain formation of the dead polymers during the polymer storage and purification process of the polymers. Owing to very slow fragmentation reaction of the TPPT-terminated polymethacrylate radical and addition reaction of this radical with a primary radical, the growing chain radicals are difficult to be regenerated, leading to an unobvious change of the molecular weight with monomer conversion. The molecular weights of polymers can be controlled by the ratios of monomer/initiator and TPPT/initiator. However, the first order kinetics of the polymerization and the polymers with narrow polydispersity are obtained, and these phenomena are discussed. This study provides useful information on how to design a better controlling agent.

## 1. Introduction

In the past two decades, controlled radical polymerization has revolutionized and revitalized the field of synthetic polymer chemistry, fruitful achievements have been gained and a number of polymerization strategies were devised to control free radical polymerization process [1,2,3]. All of these synthetic strategies afford living characters by lowering the concentration of a growing radical species, which is generally achieved by introducing a population of dormant chains that is much higher in concentration relative to the growing chain radicals [4,5]. With different synthetic strategies, creation of the dormant chains and their equilibrium with the propagating chain radicals is different. For atom transfer radical polymerization (ATRP), the dormant chain is a halide-terminated polymer (P_i_-Br) that exists in fast equilibrium with the propagating chain radicals in the presence of cuprous bromide/coordinative ligand as shown in reaction equation 1 [6,7,8,9,10,11]. The dormant chain in the nitroxide mediated radical polymerization (NMP) is nitroxide-terminated polymer chain that exists in equilibrium with a growing chain radical (reaction Equation (2)) [12,13,14,15,16,17]. Different from equilibrium reactions in ATRP and NMP, there is an equilibrium reaction as shown in reaction Equation (3) in the reversible addition-fragmentation chain transfer (RAFT) polymerization [18,19,20,21].


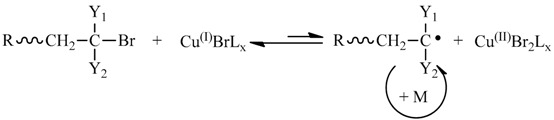
(1)


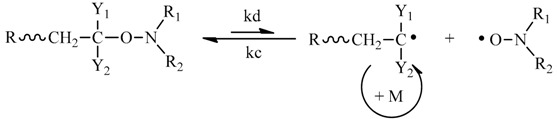
(2)


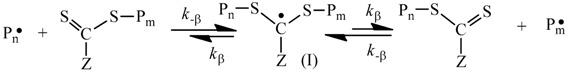
(3)

Besides the above well-known three equilibrium reactions, there are some other reactions applied in the controlled radical polymerization [4,22,23,24,25,26,27,28]. Among these controlled radical polymerizations, the thioketone-mediated radical polymerization received our attention because it was reported that the mechanism is different from ATRP and RAFT polymerization, and is an inversion of the NMP mechanism [28,29]. The living characteristics of the polymerization are attributed to reversible addition of growing chain radicals onto the thioketone spin trap as shown in reaction Equation (4), and this reaction significantly reduces the concentration of propagating free radicals due to formation of a highly stabilized chain radical (dormant radical) [29]. Due to irreversible termination between the propagating chain radicals and the dormant thioketone radicals, control of the molecular weights and narrow polydispersity indices of the yielded polymers are not as good as ATRP and RAFT polymerizations [29,30]. If the thioketone radical is more stable, can the irreversible terminations be reduced? So, reconsidering the structure of thioketone agent is necessary for answering this interesting question.


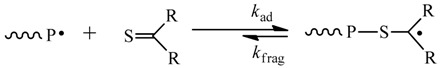
(4)

In design of the thioketone agents, two factors we considered are the adduct radical center with high stabilization and the highly steric hindrance for providing an obstacle toward biradical termination [5]. By combining high trapping free radical efficiency of the thioketone and resonance of the allylic radical, a novel control agent, 1,3,3-triphenylprop-2-ene-1-thione (TPPT) was designed and then used to mediate free radical polymerization. The resonance structure of an allylic radical is well known [31,32], generally, this radical is formed by transfer reaction of the propagating radicals with allyl compounds in the radical polymerizations owing to weakness of the allylic C–H bond [33], however, this transfer reaction is not expected in the controlling polymerization because a large quantity of dead polymer chains are produced. So, conjugation of the thione to the CH=CH_2_(S=C–CH=CH_2_) is requisite because the C=S bonds are considerably weaker than C=C bonds due to considerably small singlet-triplet excitation energies of the former bonds [34,35], so, the growing radicals are rapidly trapped by the TPPT to form allylic radicals [5,35]. The radical III in reaction Equation (5) is highly stable and cannot initiate the monomer to polymerize. Although the allylic radical is of high resonance stability, allyl bromide was successfully used as initiator in the ATRP of styrene [36]. This problem can be solved by substitution of two phenyl groups to the allylic CH_2_ (II in reaction Equation (5)) because the *π* accepting substituent can delocalize and therefore stabilize the unpaired electron, and the steric hindrance of two phenyl groups may be another reason. 


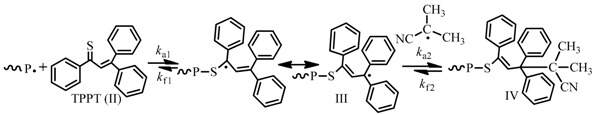
(5)

When TPPT is used as a control agent in the free radical polymerization, theoretically, there is an equilibrium reaction as shown in reaction Equation (5), and the formed intermediate radical III is highly stabilized by two phenyl moieties and one allylic substituent, which is more stable than the chain radical in reaction Equation (4). Owing to high steric hindrance of the chain radical III, its self-termination and cross-termination reactions of the chain radical III with the propagating radicals may be significantly reduced, however, its termination with primary radicals may occur to form dormant chain IV owing to less steric hindrance of the primary radical relative to polymethacrylate radicals. Even so, this chain IV is thermally unstable and its thermal homolytic dissociation should occur at a certain temperature as shown in reaction Equation (5). In order to clarify whether the equilibrium reaction 5 occurs and how this reaction affects the polymerization, we studied the polymerization behaviors and possible polymerization mechanism of methacrylates including methyl methacrylate (MMA), butyl methacrylate (BMA), benzyl methacrylate (BzMA), glycidyl methacrylate (GMA), (2-dimethylamino)ethyl methacrylate (DMAEMA), and 2-(*tert*-butylamino)ethyl methacrylate (*t*BAEMA).

## 2. Materials and Methods

### 2.1. Materials

2,4-Bis (4-methoxyphenyl)-1,3,2,4-dithiadiphosphetane 2,4-disulfide (Lawesson’s reagent) and 1,1,3-triphenyl-2-propyn-1-ol were purchased from TCI (Tokyo, Japan), both were used as received. Methyl methacrylate (MMA, 99%), glycidyl methacrylate (GMA), butyl methacrylate (BMA) and benzyl methacrylate (BzMA) were all purchased from Shanghai Chem. Co. (Shanghai, China) and purified by vacuum-distilled, and then stored at −18 °C prior to use, The 2-(*tert*-butylamino)ethyl methacrylate (*t*BAEMA) and (2-dimethylamino)ethyl methacrylate (DMAEMA) were purchased from Sigma-Aldrich (Shanghai, China) and purified by passing through an alumina column to remove the inhibitor before polymerization. Azobis(isobutyronitrile) (AIBN, Sigma-Aldrich, Shanghai, China) and dimethyl 2,2′-azobis(2-methylpropionate) (AIBME, Energy Chemical, Shanghai, China) were respectively recrystallized from ethanol. All other solvents with analytical grade were purchased from Shanghai Chemical Reagent Co. (Shanghai, China) and used without purification. 

### 2.2. Characterizations

^1^H NMR and ^13^C NMR spectra were acquired on a Bruker 400 MHz spectrometer in CDCl_3_. Molecular weight and *M*_w_/*M*_n_ were determined by a Waters 150C gel permeation chromatography (GPC) (Waters, Milford, MA, USA) equipped with microstyragel columns and RI 2414 detector (Waters, Milford, MA, USA) at 35 °C, monodispersed polystyrene standards were used in the calibration of *M*_n_, *M*_w_, and *M*_w_/*M*_n_, and THF (or DMF) was used as eluent at a flow rate of 1.0 mL/min. 

The matrix-assisted laser desorption ionization time-of-flight mass spectrometry (MALDI-TOF MS) measurements were conducted on a Perspective Biosystem Voyager-DESTR MALDI-TOF Mass spectrometer (Atouflex Speed) (Bruker, Karlsruhe, Germany). A matrix solution of 2-[(2E)-3-(4-*tert*-butylphenyl)-2-methylprop-2-enylidene]malononitrile (DCTB) (20 mg/mL in CH_2_Cl_2_) and the polymer solution (10 mg/mL in CH_2_Cl_2_) were mixed in a ratio of 2:1 (*v*/*v*). Sodium trifluoroacetate was prepared in methanol at 1 mg/ mL.

The electron paramagnetic resonance (EPR) measurements were performed on a JES-FA200 X-band spectrometer (JEOL, Tokyo, Japan) equipped with an ES-HTXA high-temperature cavity (JEOL, Tokyo, Japan), which is capable of varying the temperature from room temperature (293 K) to the maximum of 873 K. Stock solution composed of freshly distilled MMA (30.5 mg, 3.04 × 10^−4^ mol), AIBN (5 mg, 3.04 × 10^−5^ mol) and TPPT (18.3 mg, 6.08 × 10^−5^ mol) in toluene (0.2 mL) was prepared, and the solution was carefully transferred into quartz EPR tube and degassed by purging with argon for 5 min. The EPR measurements were performed by placing the tube into the preheated cavity of the ESR spectrometer at 70 °C. 

### 2.3. Synthesis of 1,3,3-Triphenylprop-2-ene-1-thione (TPPT)

A general procedure for preparation of the TPPT is as follows: 1,1,3-triphenyl-2-propyn-1-ol (4.26 g, 0.015 mol) and 2,4-bis(4-methoxyphenyl)-1,3,2,4-dithiadiphosphetane-2,4-disulfide (12.12 g, 0.03 mol) were dissolved in 100 mL toluene. The mixture was stirred at 80 °C for 24 h. After filtration, the filtrates were washed with 10% NaOH aqueous solution and water, the organic layer was dried with Na_2_SO_4_ over night, and the solvents were removed under reduced pressure to yield a black viscous liquid. The crude product was isolated by the silica column chromatography (dichloromethane:hexane = 1:12). A crystalline yellow green solid was obtained in yield of 66.3% (2.98 g). ^1^H NMR (400 MHz, CDCl_3_, δ, ppm): 6.51 (s, 1H, =CH), 7.26–7.37 and 7.7.49–7.53 (*d*, 15H, 3C_6_H_4_); ^13^C NMR (100 MHz, CDCl_3_, δ, ppm): 125.4, 127.2, 127.3, 127.6, 127.7, 128.4, 128.7, 129.3, 133.1, 143.0, 143.4; MS: [M − H]^+^ = 301.42, found at 301.1064.

### 2.4. Reaction of TPPT with AIBN

The TPPT (1.2 g, 4 mmol), AIBN (0.984 g, 6 mmol) was dissolved in toluene (10 mL), and then the solution was added into 20 mL reaction tube. After degassed through three freeze-pump-thaw cycles, the reaction tube was sealed by flame under vacuum, and the sealed tube was immersed into an oil bath at 65 °C. After 24 h, the reaction was stopped by cooling. The solvent was removed under reduced pressure. The crude product was isolated by silica column chromatography using ethyl acetate: hexane (1:20, *v*/*v*) as eluent. A yellow solid compound of 0.256 g was obtained in yield of 21.3%.

### 2.5. Polymerization of MMA in the Presence of TPPT

Typical polymerization process of MMA in the presence of TPPT and AIBN was as following: MMA (487.80 mg, 4.87 mmol), TPPT (14.64 mg, 0.0488 mmol), AIBN (4 mg, 0.0244 mmol) and anisole (526.87 mg) were added in 3 mL polymerization tube. After three freeze-pump-thaw cycles, the tube was placed in a preheated oil bath at 70 °C for 12 h. The PMMA was isolated by precipitation in the methanol, followed by filtration and then dried under vacuum overnight. The final conversion (39.45%) was determined by ^1^H NMR.

### 2.6. Chain Extension of the PMMA with GMA

The first step for chain extension is preparation of the PMMA-TPPT. A typical polymerization procedure is as follows, MMA (217.4 mg, 2.17 mmol), TPPT (26.1 mg, 0.0868 mmol), AIBME (10.0 mg, 0.0434 mmol) and anisole (253.5 mg) were respectively added into two 5 mL polymerization tubes. The tubes were respectively sealed under vacuum after three freeze-pump-thaw cycles, and the sealed tubes were immersed in an oil bath at 70 °C while stirring for 24 h. The two polymerizations in two tubes were separately treated for chain extension reaction.

A solution containing GMA (617.3 mg, 4.34 mmol) and anisole (1.7783 g) was added into one of the two polymerization tubes. After three freeze-pump-thaw cycles, the tube was sealed under vacuum and the sealed tube was immersed in an oil bath at 70 °C while stirring for another 12 h. The block copolymer, PMMA-*b*-PGMA was obtained by precipitation in the methanol, followed by filtration and then dried under vacuum overnight.

The polymerization solution in another vessel was put into the methanol while stirring to precipitate the PMMA, and the pure PMMA was obtained by filtration and then dried under vacuum overnight. The final conversion (47.3%) was determined by ^1^H NMR. The obtained PMMA (20 mg), GMA (617.3 mg, 4.34 mmol) and anisole (1.507 g) were added in 5 mL reaction tube. The tube was sealed under vacuum after three freeze-pump-thaw cycles, and the sealed tube was immersed in an oil bath at 70 °C while stirring for 12 h. The PMMA-*b*-PGMA was obtained by precipitation in the methanol, followed by filtration and then dried under vacuum overnight.

### 2.7. Polymerization Kinetics Study

The GMA (3.0865 g, 0.0217 mol), TPPT (0.1305 g, 0.434 mmol), and AIBME (49.91 mg, 0.217 mmol) were mixed in the anisole (3.0865 g) while stirring until transparent solution was formed. The solution was equally divided into five portions; each portion was placed in a 5 mL polymerization tube. The tubes were separately sealed under vacuum after three freeze-pump-thaw cycles, and the five sealed tubes were immersed in an oil bath at 70 °C, and the polymerization stopped respectively at 1, 2, 4, 6, and 12 h of polymerization by rapidly cooling the tubes to room temperature. An aliquot of the polymerization solution was taken for ^1^H NMR measurements, and the remained solution was dropped into the methanol while stirring, the precipitated PGMA was collected by filtration and then dried under vacuum overnight.

For MMA polymerization kinetic study, MMA (3.4784 g, 0.0348 mol), TPPT (0. 2085 g, 0.694 mmol), and AIBME (79.81 mg, 0.347 mmol) were mixed in anisole (3.48 g) while stirring until the solution became transparent. The solution was divided into eight portions, each portion was added into 5 mL polymerization tube and the other procedure is the same with the GMA polymerization kinetic study.

## 3. Results and Discussion

The TPPT was synthesized by reaction of 1,1,3-triphenyl-2-propyn-1-ol with the Lawesson’s reagent in the yield of 66.3% as shown in Scheme S1, and its ^1^H NMR and ^13^C NMR spectra in Figure S1 support its structure. The obtained compound was used as controlling agent in the following studies.

### 3.1. Polymerization Kinetics

**Figure 1:**
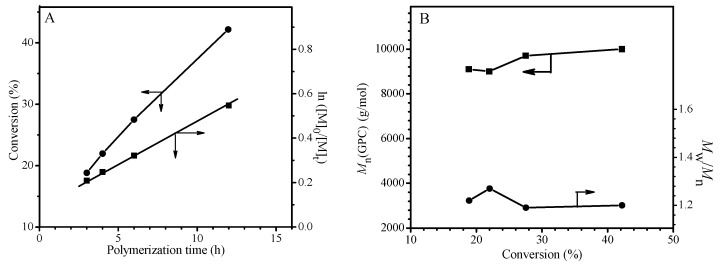
Polymerization kinetics (**A**) and relationship of *M*_n_(GPC) and *M*_w_/*M*_n_ with conversion (**B**) for the polymerization of Methyl methacrylate (MMA) with the feed molar ratio of MMA/TPPT/AIBME = 100/2/1 in anisole (50 wt %) at 70 °C.

Generally, the first order kinetics, linear increase of the molecular weight with the monomer conversion and the low polydispersity indices (less than 1.5) are the characteristics of a controlled radical polymerization. Thus, ^1^H NMR technique was used to follow the monomer conversions in the polymerization of MMA with feed molar ratio of MMA/TPPT/AIBME = 100/2/1 at 70 °C, and the results are shown in Figure 1. Similar to the kinetics of controlled radical polymerization, the monomer conversions gradually increase with reaction time, and the plot of ln([M]_0_/[M]_t_) against polymerization time displays a linear relationship, demonstrating the constant chain radical concentration in the polymerization (Figure 1A). However, the straight line does not pass through the original point (Figure 1A), which implies that the initial polymerization rate is faster before 3 h of polymerization (see Figure S2A) probably due to high concentration of the primary radicals formed through the AIBME decomposition at initial stage of polymerization and relatively slow capture of the chain radicals by TPPT.

**Figure 2:**
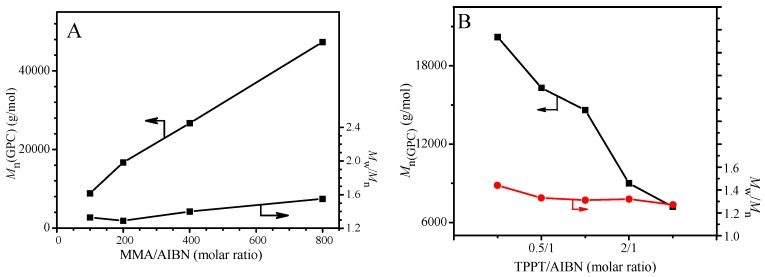
Influences of MMA/AIBN (**A**) and TPPT/AIBN (**B**) ratios on the molecular weight and *M*_w_/*M*_n_ of the polymers obtained from the polymerization with feed molar ratios of TPPT/AIBN = 2/1 (**A**) and MMA/AIBN = 100/1 (**B**) in anisole at 70 °C for 12 h. (Monomer content: 50 wt %).

In order to test other two characteristics of the livingness, the GPC was used to follow the polymerization of MMA in the presence of TPPT and the results are shown in Figure 1B. The plots of the *M*_n_(GPC) and *M*_w_/*M*_n_ against conversion in Figure 1B display relatively high molecular weight of the resultant PMMA at the beginning of polymerization and almost no variation of the molecular weight with the conversion, which is attributable to hybrid behavior in the previous report [37]. Because of high stability of the chain radical III, the addition reaction of the propagating radicals with the TPPT is more rapid relative to its reversible reaction (very low *k*_f1_ in reaction Equation (5)), so, obvious regeneration of the growing chain radicals is not observed. In addition, due to high steric hindrance of both the chain radical III and the growing PMMA radical, the cross-termination of the chain radical III with the primary radical is absolutely predominant in the competition reactions of the radical III with the growing PMMA radical or the primary radical. Reversible reaction of the dormant chains IV yields the chain radicals III and the primary radicals, the latter initiates the polymerization of MMA forming new chains or reacts with the radical III, so, regeneration of the growing chain radical is impossible. As we know, in the controlled radical polymerizations, prerequisite for controlling over the chain growth is regeneration of the growing chain radicals. Therefore, this unusual behavior is attributed to high stability and high steric hindrance of the chain radical III leading to termination of the chain propagation and almost immovability of the molecular weight with monomer conversion. Although relationship between the molecular weight and the conversion is similar to the conventional radical polymerization, polydispersities of the resultant PMMAs are relatively narrow and their polydispersity indices vary in between 1.19 and 1.27 during 24 h of polymerization (Figure 1B and Figure S2B). The polydispersity difference from the conversional radical polymerization is probably due to a different polymerization mechanism. In the conventional radical polymerization, the molar ratio of monomer to initiator is a main factor influencing the chain length, however, the main factor in the present system is molar ratio of [monomer]/[TPPT]/[initiator] when the temperature is fixed. As shown in Figure 2A, molecular weights of the obtained PMMA increase from 8800 g/mol to 47,000 g/mol when the molar ratios of MMA/AIBN increase from 100 to 800 because this ratio increases, it infers the decrease of AIBN and TPPT concentrations in the monomer solution, which leads to an increase of the PMMA chain growth rate. The molar ratio of TPPT/AIBN also affects the molecular weights of polymer obtained. When the molar ratios of TPPT/AIBN increases from 0.25 to 4, the molecular weights of PMMA formed decrease (Figure 2B) because this ratio increase will increase the possibility of PMMA radicals terminated by TPPT, reducing the life time of growing chain radicals.

**Figure 3:**
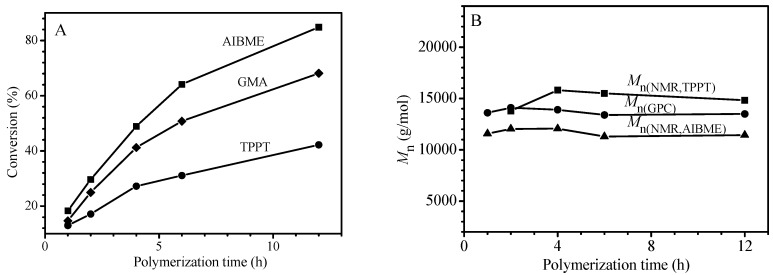
The relationship of the GMA, AIBME and TPPT conversions with polymerization time (**A**) and the dependence of molecular weights (*M*_n_s) measured by NMR method and gel permeation chromatography (GPC) method (**B**) for the polymerization of GMA with feed molar ratio of GMA/TPPT/AIBME = 100/2/1 at 70 °C in anisole (50 wt %).

The results in Figure 2 demonstrate that the main factors influencing chain length of the formed polymer are the concentrations of AIBN and TPPT in the monomer and their ratio. To better understand why the polymer is of narrow polydispersity, the ^1^H NMR was used to follow the polymerization of glycidyl methacrylate (GMA), not MMA using AIBME, not AIBN as initiator because the ester methyl proton signal of the AIBME can be well isolated in ^1^H NMR spectra of the polymerization solutions, and Figure S3 is ^1^H NMR spectra of the polymerization solutions obtained at different polymerization time. The proton signals at δ = 6.52 (a), 6.17 (c) 5.62 (d), and 3.69 ppm (b) are respectively ascribed to vinyl proton of the TPPT, vinyl protons of the GMA and ester methyl protons of the AIBME. Based on integral values of these proton signals obtained at different polymerization time, the conversions of TPPT, GMA, and AIBME were calculated, the plots of their conversions against reaction time are shown in Figure 3A, and the disappearance rate of AIBME is consistent with the first order kinetics as shown in Figure S4 and the *k*_d_ at 70 °C is 4.42 × 10^−5^ s^−1^.

Figure 3A reveals that disappearance rates of the three components in the polymerization system have the following order: AIBME > GMA > TPPT, which is different from RAFT polymerization. In the latter case, the primary radicals are produced not only from homolysis of the initiator, but also from chain transfer reaction of the RAFT agent, leading to fast disappearance of the initiator and the RAFT agent. Comparatively, in the present system, the primary radical is produced only from homolysis of AIBME, resulted in slow disappearance rates of AIBME and very slow disappearance rate of TPPT. As shown in Figure 3A, after 12 h of polymerization, the AIBME, the GMA and the TPPT remain 15.2%, 31.9%, and 57.8% of their original dosages, respectively, their concentrations and ratio of GMA/TPPT/AIBME are obviously changed with the reaction time. For example, the molar ratios of TPPT/AIBME remained in the polymerization system increase from 2/1 to 7.6/1 when the polymerization starts from the beginning to 12 h of polymerization. This result is reasonable when we consider that homolysis of one AIBME molecule must consume one TPPT molecule, theoretically (see reaction Equation (5)), which must result in more TPPT remained in the reaction system than the AIBME, however, the most important factors influencing the chain length are life time and propagating rate of the growing radicals, and the molecular weight is controlled mainly by the ratios of TPPT/growing radicals and GMA/growing chain radicals. As we know, the growing chain radicals are formed through initiation of the primary radicals, and the primary radicals existed in the polymerization system come not only from homolysis of the initiator but also from regeneration of the dormant chain IV (see reaction Equation (5)). Because more and more dormant chains are accumulated with polymerization time, the primary radicals produced from regeneration of the dormant chains display the same trend, which is different from the conventional radical polymerization. This leads to no obvious change of the growing chain radicals during the polymerization process as shown in Figure 1A, although the initiator concentration significantly decreases with progress of the polymerization (Figure 3A). In addition, the polymerization system becomes viscous with accumulation of the formed polymer; the viscous solution reduces the termination rate of the growing chain radicals by TPPT. All these factors bring about similar chain lengths of the formed polymers during the polymerization as shown in Figure 1B. 

In order to verify the reliability of reaction Equation (5), the number-average molecular weights (*M*_n_s) of the formed PGMA chains were calculated based on the data showed in Figure 3A. Assume that one AIBME molecule cleaved or one TPPT molecule consumed can form one PGMA macromolecule, and the initiator efficiency (*f*) is 100%, *M*_n_s of the formed polymer can be calculated based on the conversions of GMA, AIBME, and TPPT, more specifically, based on the molar ratios of GMA converted/AIBME converted [*M*_n(NMR,AIBME)_], or GMA converted/TPPT converted [*M*_n(NMR,TPPT)_]. Figure 3B shows the relationship of the *M*_n_(GPC), *M*_n(NMR,AIBME)_,and *M*_n(NMR,TPPT)_ with polymerization time. Although the *M*_n(NMR,AIBME)_ is less than the *M*_n(NMR,TPPT)_ due to *f* < 100%, the two values are close to the *M*_n_(GPC) that is in the middle of the two *M*_n(NMR)_s (see Figure 3B). So, this result is consistent with these reactions in reaction Equation (5): one primary radical initiates GMA to polymerize; the formed growing chain radical reacts with one TPPT; and the formed radical III is terminated by other one primary radical.

**Figure 4:**
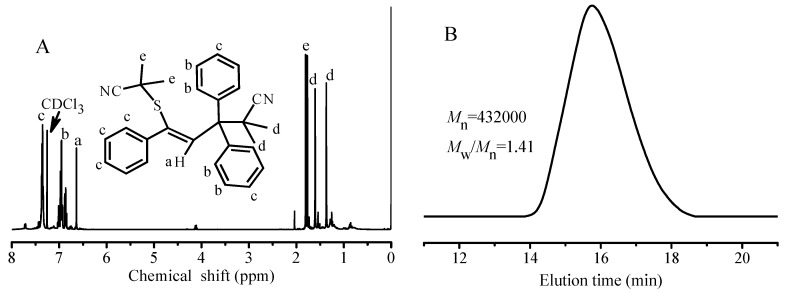
(**A**) ^1^H NMR spectrum of the reaction product between AIBN and TPPT, 1,3,3-triphenyl-1-isobutylnitilesulfo-3-isobutylnitrileprop-2-ene; (**B**) GPC curve of the PMMA obtained from the polymerization of MMA using TBBP as initiator with feed molar ratio of MMA/TBBP = 400/1 in anisole (weight ratio of MMA/anisole = 50/50) at 70 °C for 6 h.

As aforementioned, the cross-termination between the growing chain radical and the intermediate radical III did not occur, and the PGMA-TPPT radical reacts with the primary methyl 2-methylpropionate radical. In order to provide more evidences to support the above conclusion, a solution of AIBN and TPPT was heated at 70 °C for 24 h, the adduct product was obtained after silica column chromatography using ethyl acetate:hexane (1:20, *v*/*v*) as eluent, and characterizations of the resultant product fully support the structure of 1,3,3-triphenyl-1-isobutylnitilesulfo-3-isobutylnitrileprop-2-ene (TBBP, see chemical formula in Figure 4A). ^1^H NMR spectrum in Figure 4 reveals the proton signals at δ = 1.81 and 1.76, 1.59 and 1.36 ppm, which are respectively ascribed to two methyl groups of the isobutyronitrilesulfo and the isobutyronitrile groups respectively in the 1 and 3 position of the propene. The measured molecular weight is 437.21, which is very close to the theoretical value 436.61, and the ^13^C NMR spectrum in Figure S5 is also consistent with the structure of TBBP. In addition, the solution of MMA in the presence of TBBP was heated at 70 °C, after 6 h of polymerization, the GPC curve of the obtained PMMA is shown in Figure 4B. So high molecular weight indicates very slow homolysis rate of the dormant chains IV, but the reversible reaction (*k*_f2_) is really occurring. All these facts verify the reliability of addition reaction (*k*_a2_) and reversible reaction (*k*_f2_) in equilibrium reaction (5). 

**Figure 5:**
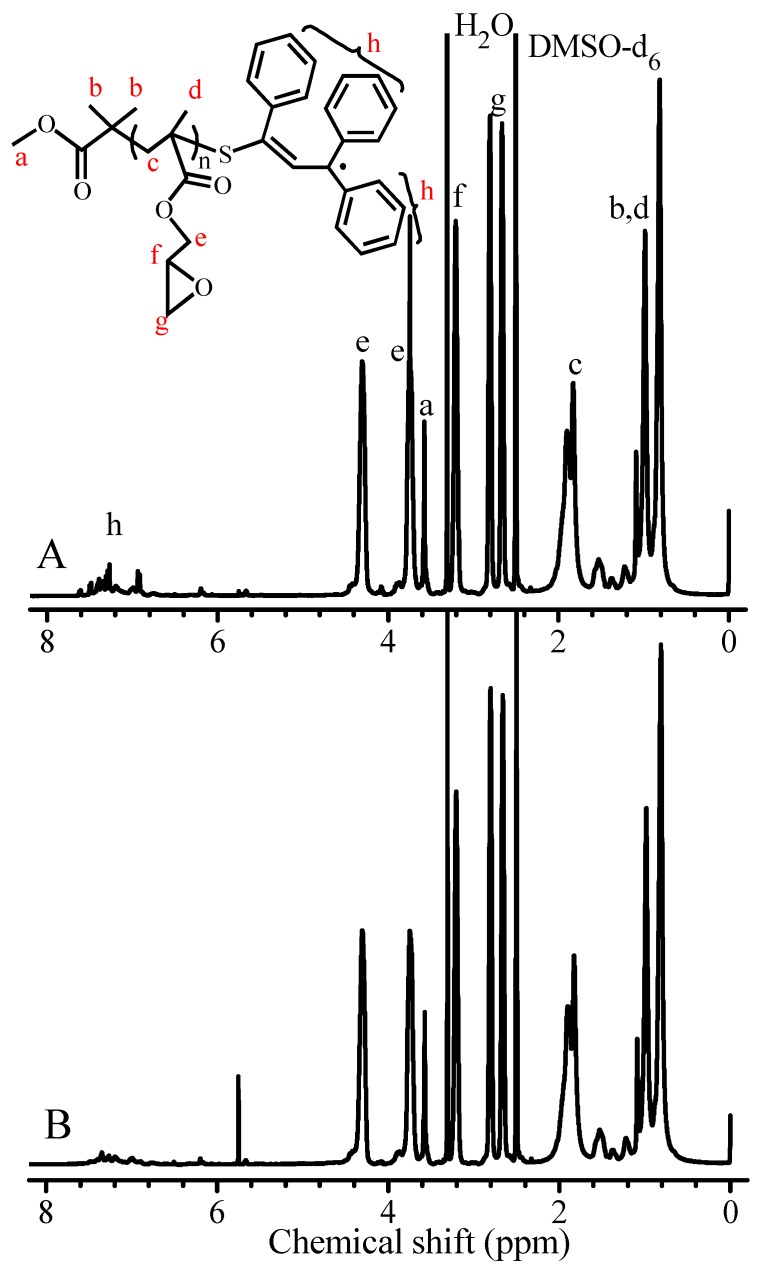
Influence of purification times on the TPPT contents of the obtained PGMA. ^1^H NMR spectra of the PGMA respectively obtained after the first (**A**) and the third (**B**) dissolution-methanol precipitation cycle of the PGMA prepared from the polymerization of GMA with the feed molar ratio of GMA/TPPT/AIBME = 10/2/1 at 70 °C for 24 h.

As aforementioned in the discussion on Figure 1B, almost no variation of molecular weight with conversion is due to negligible reversible reaction (*k*_f1_) of the intermediate chain radical III to form the growing chain radical and TPPT during the polymerization. It is necessary to clarify whether this reversible reaction (*k*_f1_) exists, if it exists, in what condition can this reaction occur? Therefore, the obtained PGMAs were characterized, and their ^1^H NMR spectra of PGMA are shown in Figure 5. The results evidence the addition reaction (*k*_a1_) of growing chain radicals with TPPT, the epoxy methylene (g), epoxy methine (f), and ester methylene (e) proton signals of the glycidyl group in PGMA respectively appear at δ = 2.79, 2.66 (g); 3.19 (f) and 4.32, 3.74 (e), the proton signals at δ = 3.58 (a) and 6.75~7.52 ppm are respectively attributed to the ester methyl protons (a) of the AIBME, aromatic and allylic protons (h) of the TPPT, ascription of other proton signals is marked in Figure 5. Existence of the isobutyronitrile and the TPPT in the PGMA chains demonstrates that the initiation of AIBME and the addition reaction between the growing chain radicals and TPPT are really taken place to form intermediate chain radical III (see reaction Equation (5)). However, when we compare ^1^H NMR spectra of the PGMAs obtained from different dissolution-precipitation cycles for purification of the polymer, we always observe decrease of the proton signals appeared in the range of 6.74 and 7.5 ppm with increase of the dissolution-precipitation cycle. Figure 5A,B are the ^1^H NMR spectra of the PGMA obtained respectively after one and three dissolution-precipitation cycles of the PGMA formed in the polymerization with the feed molar ratio of GMA/TPPT/AIBME = 10/2/1 at 70 °C for 24 h. Because the monomer unit cannot be lost during the purification, the integration ratio of proton signal e to signals h [I_e_/(I_h_/16)] is used to identify the change of TPPT content in the PGMA. For the PGMA obtained after one dissolution-precipitation (in methanol) cycle, this ratio is 55.4, that is, the TPPT content is 1.8%. However, after three dissolution-precipitation cycles, the proton signals h at 6.74~7.5 ppm obviously decrease as shown in Figure 5B [I_e_/(I_h_/16) = 86.9], and the TPPT content reduces from 1.8% to 1.1%. The TPPT content decrease implies that the equilibrium reaction 5 reversibly shifts from the intermediate chain radical III to the growing chain radical and TPPT, and the regenerated TPPT is removed during the polymer purification process, so, the TPPT content in the PGMA decreases, and more dissolution-precipitation cycles result in more low TPPT content in the polymers. Thus, the molecular weight of PGMA separated from the polymerization solution cannot be calculated based on ^1^H NMR data, more specifically, the integration ratio of proton signal e to signal h because some PGMA chains do not contain one TPPT unit due to precipitation process. Here, some reasonable questions arise, are those PGMA chains without TPPT unit produced during the polymerization and/or during the polymer purification and the storage? How can the intermediate chain radical III regenerate the growing chain radicals?

**Figure 6:**
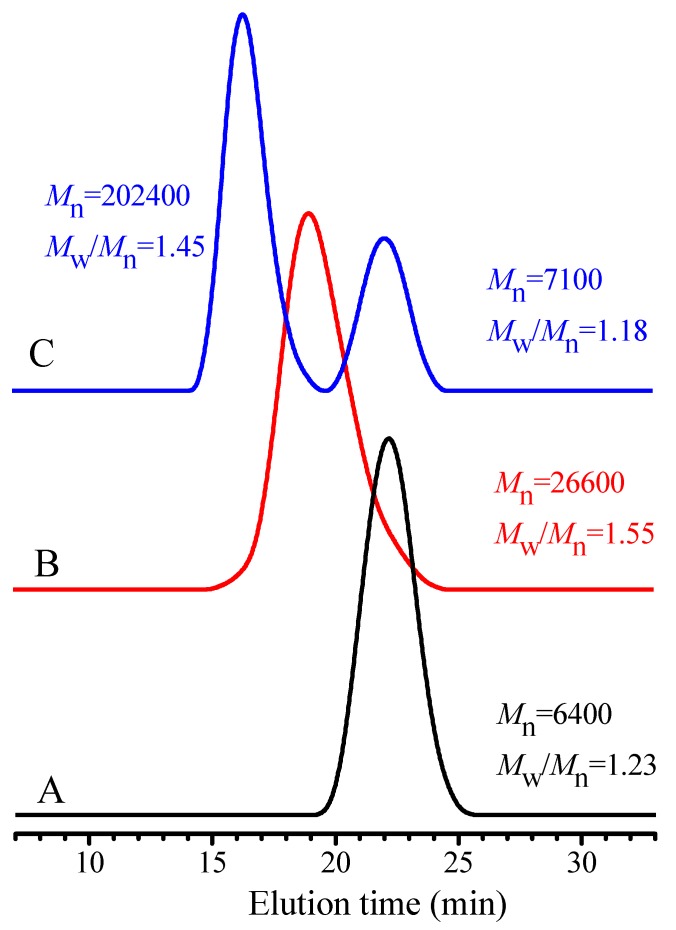
GPC traces of (**A**) the PMMA obtained from the polymerization of MMA with feed molar ratio of MMA/TPPT/AIBME = 50/2/1 in anisole (50 wt %) at 70 °C for 24 h; (**B**) chain extension polymerization of the PMMA (*M*_n_ = 6400 and *M*_w_/*M*_n_ = 1.23) was conducted by directly adding the GMA (its dosage is double moles of the MMA used) into the polymerization solution and then was carried out in anisole (30 wt %) at 70 °C for 12 h; (**C**) chain extension polymerization of the PMMA with the GMA (its dosage is double moles of the MMA used) in anisole (30 wt %) at 70 °C for 12 h using PMMA, which was obtained after one precipitation in methanol, as the extender.

In order to answer the above questions, two polymerizations of the MMA with the same feed molar ratio of MMA/TPPT/AIBN = 50/2/1 were separately carried out at 70 °C in the two reaction tubes. After 24 h polymerization, the second monomer, GMA was directly added into one reaction tube for chain extension polymerization of the formed PMMA. The polymerization in another tube was stopped and the formed PMMA was obtained by precipitation in methanol for one time. Except a small amount of PMMA was used for GPC measurement, the residual PMMA was applied as extender in the subsequent polymerization of GMA. After both extension polymerizations were conducted at 70 °C for 12 h, the block copolymers were respectively obtained by precipitation in methanol, and the GPC traces of the block copolymer and their precursor are respectively shown in Figure 6A–C.

The GPC curves of two block copolymers in Figure 6B,C are obviously different, the GPC curve of block copolymer obtained by directly adding GMA into the MMA polymerization solution is single and is shifted to the high molecular weight position, and no obvious PMMA remains (see Figure 6B). From these results, we can reasonably conclude that fragmentation reaction of the intermediate chain radicals III occurs to regenerate the growing PMMA radicals that initiate the polymerization of GMA to produce the block copolymer, PMMA-*b*-PGMA. Its structure is confirmed by its ^1^H NMR spectrum as shown in Figure S6, the proton signals for the ester methylene of PGMA and the ester methyl of PMMA respectively appear at δ = 4.31 (f), 3.81 (f) and 3.60 ppm (a), ascription of other proton signals is marked in Figure S6. Adding GMA into the polymerization solution significantly lowers concentrations of the AIBME (only 2.2% of AIBME remains at 24 h polymerization) and the growing PMMA radicals, leading to relatively fast regeneration of the growing PMMA radicals in comparison with the chain radicals produced by AIBME initiation. However, for the polymerization of GMA using the obtained PMMA as extender, the resultant polymer displays two GPC curves, one curve with *M*_n_ = 202,400 g/mol and *M*_w_/*M*_n_ = 1.45, other curve with *M*_n_ = 7100 g/mol and *M*_w_/*M*_n_ = 1.18 (Figure 6C), the latter *M*_n_ is close to the value of its precursor (*M*_n_ = 6400 g/mol, *M*_w_/*M*_n_ = 1.23). The former comes from chain extension of the active PMMA chains, and the latter is the dead PMMA formed by disproportionation termination of the PMMA chain radicals. The above results reveal that no obvious PMMA radicals are terminated through the disproportionation reaction in the polymerization due to very low concentration of the growing PMMA radicals probably because the TPPT can efficiently capture the growing radicals. The dead PMMA is mainly produced in the cease-polymerization and the purification process, probably, no new formed primary radical and dilution of the resultant PMMA benefit reversible reaction of the intermediate chain radicals III in reaction Equation (5), leading to regeneration of the growing chain radicals, and if no new monomer is added, the dead PMMA is formed through the termination reactions between the chain radicals.

### 3.2. EPR Studies

**Figure 7:**
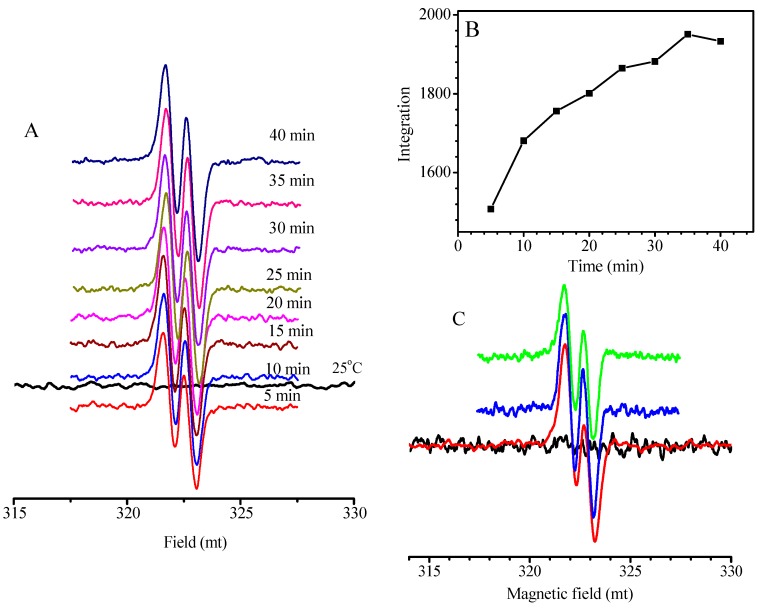
Electron paramagnetic resonance (EPR) spectra of the chain radicals produced at different reaction time (**A**) and relationship of the integration of EPR signals with the reaction time (**B**) for the polymerization with feed molar ratio of MMA/TPPT/AIBN = 10/2/1 at 70 °C; (**C**) EPR spectra of the radicals formed in the reaction of TPPT with AIBN (molar ratio of TPPT/AIBN = 2/1) at 70 °C for different reaction time.

In the aforementioned discussion, very slow reversible reaction of the chain radicals III, which leads to no change of the molecular weight with conversion, is due to very stable intermediate chain radicals III. Owing to a big rate difference between the addition reactions and their reversible reactions, the concentration of chain radicals III is relatively high; reasonably, they can be detected by electron paramagnetic resonance (EPR). Therefore, EPR spectra were used to follow the polymerization with a feed molar ratio of MMA/TPPT/AIBN = 10/2/1 at 70 °C for different reaction time, and the results are shown in Figure 7. Generally, directly probing the propagating radicals in radical polymerization by EPR is difficult mainly owing to both the labile nature and the low concentration of propagating radicals. In addition, the propagating radical produced in the MMA polymerization revealed hyperfine splitting spectrum due to couplings of the three α-methyl protons and the two β-methylene protons [38], so, the radical signal in Figure 7 cannot be ascribed to the PMMA radical and the isobutyronitrile radical formed via homolysis of the AIBN because no radical signal was detected within 1 min of polymerization. Similar to the delocalized allyl-type radicals produced in the free radical polymerizations of conjugated dienes, such as butadiene, isoprene, etc. [39], the EPR spectra in Figure 7A are assigned to the delocalized allyl-type radicals III because only this type of radical is produced in the polymerization of MMA besides the PMMA and isobutyronitrile radicals. The further evidence for this ascription is the EPR spectra shown in Figure 7C, which were obtained by using EPR technique to follow the reaction between the AIBN and the TPPT with the feed molar ratio of TPPT/AIBN = 2/1. The results, in Figure 7C, reveal almost the same radical signal shape, demonstrating both radicals are the same. Because the strength of the radical signals is proportional to their concentrations, lower integral values mean the lower concentrations of the radical III. The Figure 7B is a plot of integral values of the radical signal against polymerization time based on the results in Figure 7A, we can see that the concentrations of the chain radicals III increase fast within the initial polymerization, indicating rapid consumption of the TPPT through addition reaction of the growing chain radicals with the TPPT, and after 30 min polymerization, increase of the chain radicals III becomes slower probably owing to gradual increase of the termination reaction rate between the chain radicals III and the isobutyronitrile radicals with fast accumulation of the intermediate chain radicals III, and this reversible termination is supported by the results shown in Figure 4A. 

### 3.3. MALDI-TOF MS Studies 

**Figure 8:**
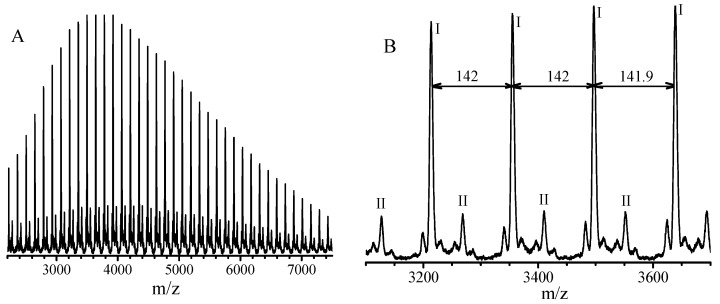
Matrix-assisted laser desorption ionization time-of-flight mass spectrometry (MALDI-TOF MS) spectrum (**A**) and enlarged spectrum (**B**) of the poly(glycidyl methacrylate) obtained by radical polymerization with feed molar ratio of GMA/TPPT/AIBN = 20/2/1 in anisole (concentration = 20 wt. %) at 70 °C for 24 h.

For the PGMA obtained after three dissolution-precipitation in methanol cycles, the number of terminal isobutyronitrile group per 100 GMA units decreases from 7.3 to 6.3 based on the ratio of I_e_/(I_a_/3) in Figure 5, this infers that the terminal group is changed during the purification process. Because the matrix-assisted laser desorption ionization time-of-flight mass spectrometry (MALDI-TOF MS) is one powerful technique to gain mechanistic insights into polymerization reactions based on the presence or absence of the mechanism-specific reaction products [40], we used this technique to study what reaction occurs during the purification process. The PGMA, instead of PMMA, was used to study its MALDI-TOF MS because the molecular weight (300.42 g/mol) of TPPT is exactly three times of the MMA molecular weight (100.12 g/mol), as a result, it is difficult to identify whether the TPPT unit exists in the obtained polymers based on the data of MALDI-TOF MS presented in Figure S7. The PGMA sample for measurement of MALDI-TOF MS was prepared by the polymerization with feed molar ratio of GMA/TPPT/AIBN = 20/2/1 in anisole at 70 °C for 24 h, the PGMA with *M*_n,_(GPC) = 4400 g/mol, *M*_w_/*M*_n_ = 1.24 was obtained, and Figure 8A is its MALDI-TOF MS, its enlarged spectrum from 3100 to 3700 g/mol is shown in Figure 8B. The peak molecular weight of the sample in Figure 8A is 3800 g/mol. We can see five series of the polymer chains that repeat with the mass of a GMA unit (142 g/mol, see Figure 8B). Among the five series, the series I is a highly abundant polymer species, and other four series exist in minor quantities. Based on the mass of the series I, the polymer chains have the structural formula: Na^+^(CH_3_)_2_(CN)C(GMA)_n-1_C_7_H_9_O_3_. When the degree of polymerization (DP) = 22, the molecular weight can be calculated to be 3214.1 g/mol, which is very close to the measured mass, 3214.6 g/mol. According to reaction Equation (5), every PGMA chain contains two isobutyronitrile groups and one TPPT; this is conflictive to the formula obtained from the data in Figure 8B, which contains only one isobutyronitrile group in each PGMA chain. During the purification process and storage of the PGMA, the reversible reaction of dormant chains IV may occur, and the disproportionation reaction between the formed two types of radicals, PGMA and isobutyronitrile radicals is possibly taken place as shown in reaction Equation (6), the formed PGMA has the formula as shown V in reaction Equation (6). This is consistent with discussion based on the data in Figure 5 and Figure 6. 


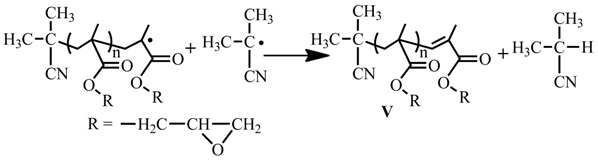
(6)

### 3.4. Polymerization of Methacrylates

To test whether the TPPT/AIBN (or AIBME) system can be used in the polymerizations of other methacrylate monomers, the polymerizations of various methacrylates in anisole were carried out at 70 °C for 12 h using TPPT and AIBN as the control agent and initiator, and the polymerization conditions and results are listed in Table 1. The GPC curves of the resultant polymethacrylates are shown in Figure S8, we can see that all polymerizations of the methacrylates listed in Table 1 can produce the corresponding polymers with relatively narrow molecular weights (*M*_w_/*M*_n_ = 1.16~1.32). However, their monomer conversions display slight difference, which is probably related to their different reactivity. Similar to the results in Figure 3A, disappearance rates of the three components for every monomer in the polymerization have the following order: AIBME > monomer > TPPT, which may be ascribed to the polymerization behaviors of these monomers similar to the GMA polymerization. The polymer structures were characterized by their ^1^H NMR spectra (Figure S9), the proton signals for the ester methylene of monomers and the ester methyl of AIBME respectively appear at δ = ~4.0 (d) and ~3.62 pp (a) and other proton signals are also ascribed as shown in Figure S9. Because the MALDI-TOF results reveal that every PGMA chain is capped with one methyl 2-methylpropionate after several dissolution-precipitation cycles (Figure 8), so, the molecular weights (*M*_n(NMR)_s) of the corresponding polymers can be calculated based on the integration ratios of proton signals d and a, as well as molecular weights of the monomers, and the results are listed in Table 1 except *M*_n(NMR)_ of the PMMA because the signal d is overlapped with the signal a (see PMMA in Figure S9). The *M*_n(NMR)_s of all polymethacrylates are lower than, but close to their corresponding *M*_n_(GPC)s besides the PBzMA. 

## 4. Conclusions

A new type of the control agent, TPPT, has been successfully synthesized, and in the free radical polymerizations, this allylthioketone compound can efficiently capture the propagating chain radicals to form TPPT-terminated chain radicals (III) that are too stable to initiate the monomer to polymerize. They (radicals III) can be reversibly terminated by the primary radicals, such as isobutyronitrile radicals, but their termination reaction with the growing polymethacrylates radicals is not observed probably owing to high steric hindrance of the growing radicals. Because the very slow fragmentation reaction of the intermediate chain radical III, leading to regeneration of the growing chain radicals impossible (see reaction Equation (5)), the polymers formed at the beginning of polymerization have relatively high molecular weight and their molecular weight is insensitive to the monomer conversion. However, the kinetic studies reveal the first order kinetics and narrow molecular weight distribution of the resultant polymers. For the polymerizations with TPPT and AIBME as control agent and initiator, the molecular weights can be controlled through feed molar ratios of the TPPT/AIBN and the monomer/AIBN. One important character for living nature of the polymerization is successful chain extension of the formed PMMA with GMA to produce the block copolymers, PMMA-*b*-PGMA by directly adding the GMA monomer into the MMA polymerization system formed after 24 h of polymerization. This demonstrates that the PMMA radicals are regenerated through fragmentation reaction of the TPPT-terminated PMMA, and then initiate the polymerization of GMA.

## Figures and Tables

**Table 1:** Conditions and results for the controlled radical polymerization of methacrylates in the presence of TPPT and AIBN in anisole (concentration: 50 wt %) ^a^.

No	Monomer ^b^	Feed molar ratio of M/TPPT/AIBN	Conversion (%) ^c^			*M*_n(NMR)_^d^ (g/mol)	*M*_n_(GPC) ^e^ (g/mol)	*M*_w_/*M*_n_^e^
			M	AIBME	TPPT			
1	MMA	100/2/1	39.9	82.9	44.5	−	11,400	1.16
2	BzMA	100/2/1	57.9	79.0	35.2	23,460	20,500	1.26
3	BMA	100/2/1	39.9	85.6	50.3	14,090	15,600	1.25
4	DMAEMA	100/2/1	37.2	81.8	40.0	12,050	14,500	1.32
5	*t*-BAEMA	100/2/1	51.5	72.0	50.6	25,380	31,000	1.26

^a^ Temperature: 70 °C; reaction time: 12 h; solvent: anisole; concentration: 50%; ^b^ MMA: methyl methacrylate; BzMA: benzyl methacrylate; BMA: butyl methacrylate; *t*-BAEMA: 2-(*tert*-butylamino)ethyl methacrylate; DMAEMA: (2-dimethylamino)ethyl methacrylate; ^c^ Conversions of monomer (M), AIBME and TPPT were measured by ^1^H NMR method; ^d^ Determined based on the integration ratios of the proton signal of ester methylene in the monomers (except PMMA) to the proton signal of ester methyl of the residual AIBME attached the polymers and the molecular weights of monomer. The molecular weight of PMMA was not obtained owing to proton signal overlapping of the ester methyl in the MMA units and the ester methyl in the residual AIBME; ^e^ Determined by GPC calibrated with polystyrene standards, for PMMA, PBzMA, and PBMA, the THF was used as eluent; for DMAEMA and *t*-BAEMA and DMF was used as the eluent.

## Supplementary Materials

The following are available online at www.mdpi.com/2073-4360/9/11/608/s1.
