# Supplementary material for: Allylthioketone Mediated Free Radical Polymerization of Methacrylates
Source: Polymers (Basel). 2017 Nov 13;9(11):608. doi: 10.3390/polym9110608 (PMC6418703; doi:10.3390/polym9110608)
Supplement: Supplementary file 1 [file polymers-09-00608-s001.pdf]

Supporting information

Allylthioketone Mediated Free Radical Polymerization of Methacrylates

Feng Zhong[1], Liang Qiu[2], Chun-Yan Hong[1]*, Cai-Yuan Pan[1]*

[1]CAS Key Laboratory of Soft Matter Chemistry, Department of Polymer Science and Engineering, University of Science and Technology of China, Hefei, Anhui, 230026, P. R. China

[2]Institute of Biophysics, Hebei University of Technology, Tianjin 300401, P.R. China

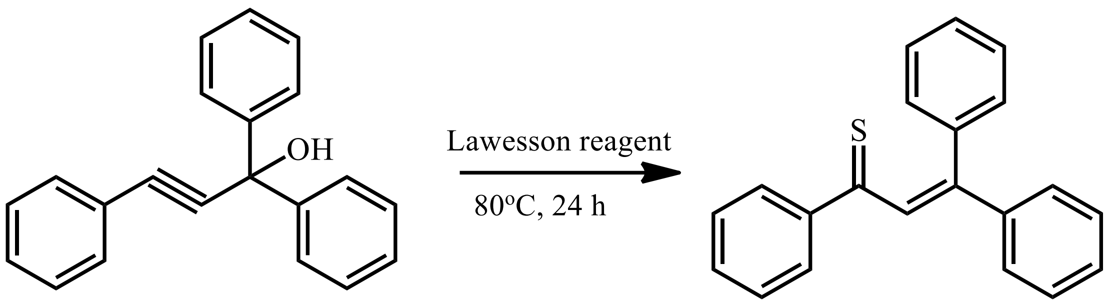

Scheme S1. Synthesis of 1,3,3-triphenylprop-2-ene-1-thione

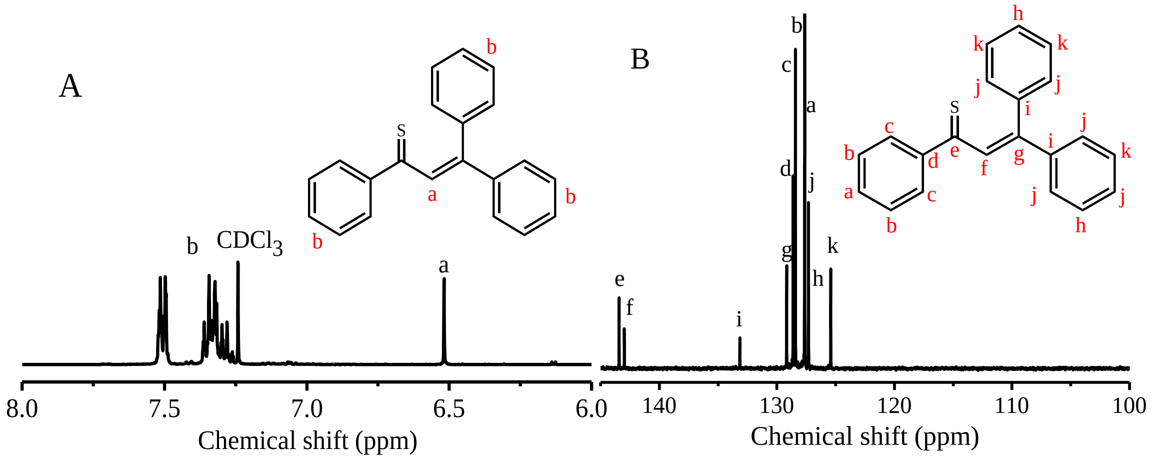

Figure S1. ^{1}H NMR (A) and ^{13}C NMR (B) spectra of 1,3,3-triphenylprop-2-ene-1-thione (TPPT)

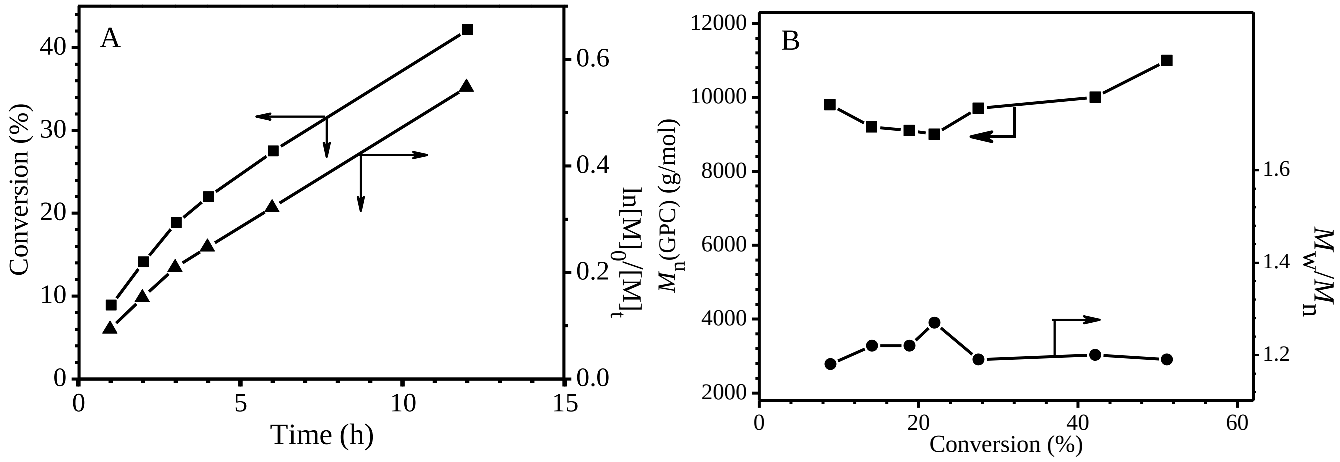

Figure S2. Polymerization kinetics (A) and relationship of M_n (GPC) and M_w/M_n with the conversion (B) for the polymerization in anisole (50 wt%) with feed molar ratio of MMA/TPPT/AIBME = 100/2/1 at 70°C.

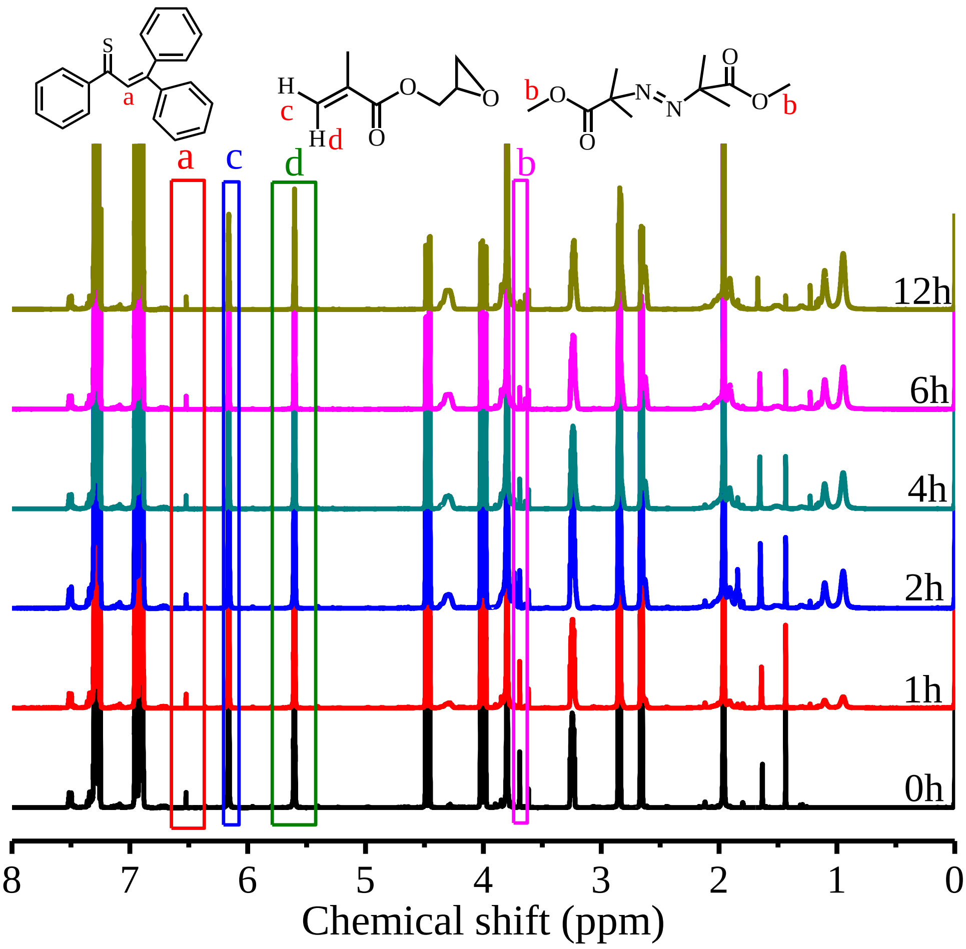

Figure S3. ^{1}H NMR spectra of the polymerization solutions for the polymerization of GMA with feed molar ratio of GMA/TPPT/AIBN=100/2/1 at 70°C in anisole (50 wt%) for different reaction time.

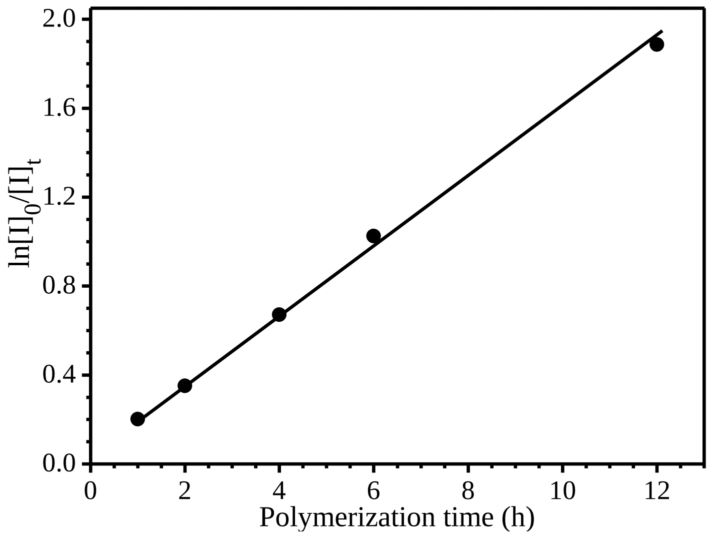

Figure S4. Kinetics of AIBME decomposition in the polymerization of GMA with the feed molar ratio of GMA/TPPT/AIBME=100/2/1 in anisole (50 wt%) at 70°C.

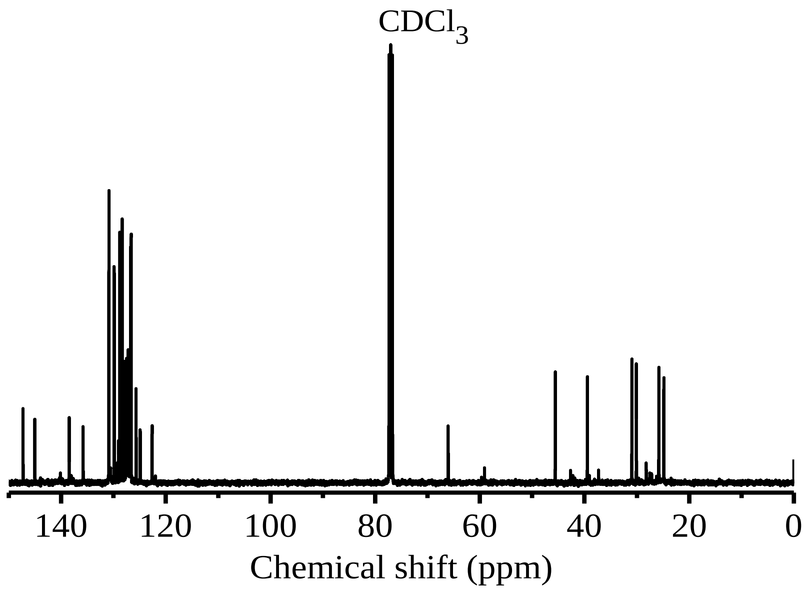

Figure S5. ^{13}C NMR spectrum of 1,3,3-triphenyl-1-isobutylnitilesulfo-3- isobutylnitrileprop-2-ene.

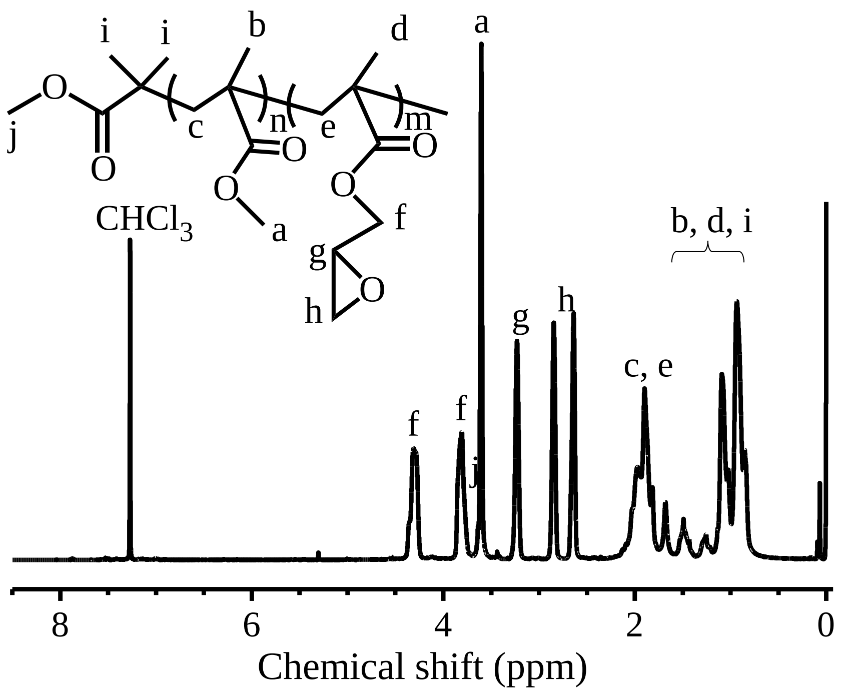

Figure S6. ^{1}H NMR spectrum of the block copolymer, PMMA-*b*-PGMA obtained from the extension polymerization of PMMA (M_n=6400 and M_w/M_n=1.23) by directly adding the GMA (double moles of MMA) into the PMMA solution, which was obtained by the polymerization of MMA with feed molar ratio of MMA/TPPT/AIBME=50/2/1 in anisole (50 wt%) at 70°C for 24 h, and then continuous polymerization in anisole (30 wt%) at 70°C for 12 h.

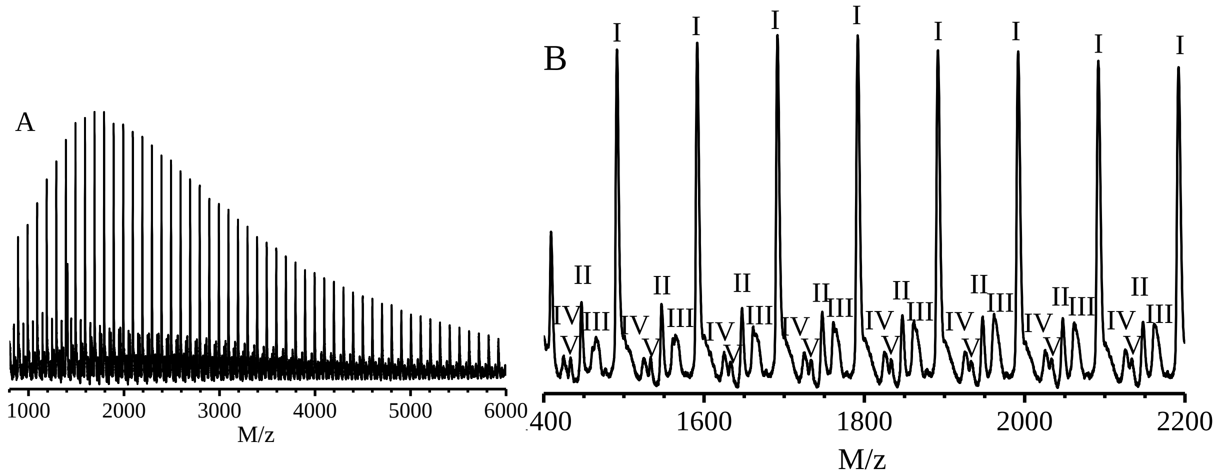

Figure S7. MALDI-TOF spectrum (A) and enlarged spectrum (B) of the poly(methyl methacrylate) obtained by radical polymerization with feed molar ratio of MMA/TPPT/AIBN=20/2/1 in anisole (concentration = 20%) at 70°C for 24h.

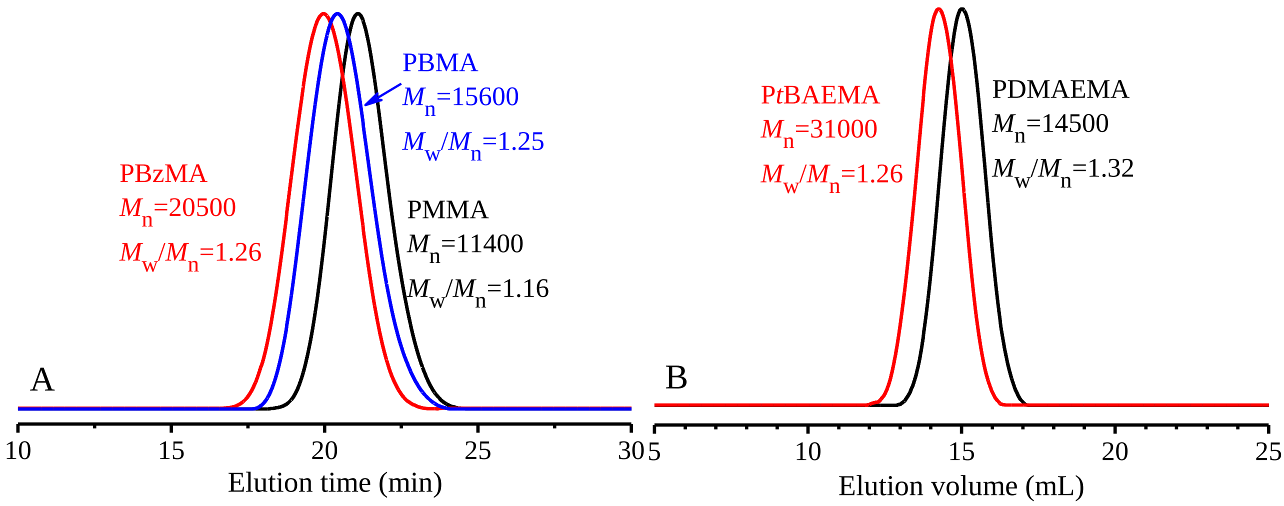

Figure S8. GPC traces of the PMMA, PBMA, PBzMA, PDMAEMA and P*t*BAEMA prepared by the polymerization with feed molar ratio of M/TPPT/AIBME=100/2/1 in anisole (50 wt%) at 70°C for 12 h. For PMMA, PBMA and PBzMA, THF was used as eluent, for PDMAEMA and P*t*BAEMA, DMF was used as eluent.

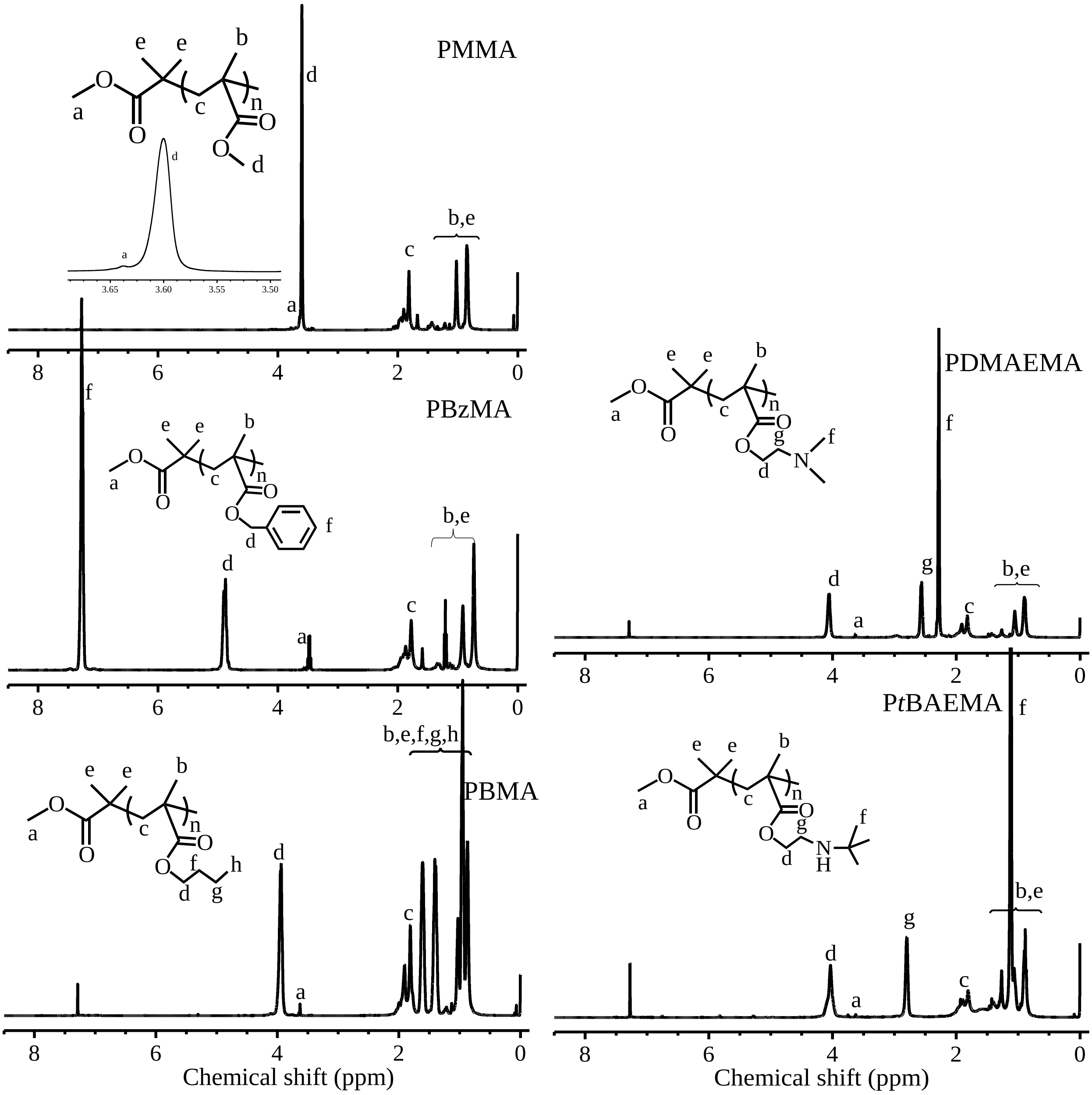

Figure S9. ^{1}H NMR spectra of PMMA, PBzMA, PBMA, PDMAEMA and P*t*BAEMA prepared by the polymerizations with feed molar ratio of M/TPPT/AIBME = 100/2/1 in anisole (50 wt%) at 70°C for 12 h.
